# Association of hyperemesis gravidarum severity with HALP score and hematologic inflammatory markers

**DOI:** 10.1038/s41598-025-20486-9

**Published:** 2025-10-17

**Authors:** Cenk Soysal, Ceren Bilir, Ahmet Burak Zambak, Onur İnce, Yasemin Taşçı

**Affiliations:** 1 Department of Obstetrics and Gynecology, Kütahya Health Sciences University, Kütahya, Turkey; 2 Department of Obstetrics and Gynecology, Hacettepe University, Ankara, Turkey

**Keywords:** Hyperemesis gravidarum, HALP score, Systemic immune-inflammation index, Neutrophil-to-lymphocyte ratio, Pregnancy, Biomarkers, Diseases, Immunology, Medical research

## Abstract

This study aimed to investigate the diagnostic and prognostic value of the HALP score and hematological inflammatory indices in pregnant women with hyperemesis gravidarum (HEG). In this prospective observational study, 48 pregnant women diagnosed with HEG and 51 healthy, gestational age-matched controls were enrolled. Demographic, clinical, and laboratory data were collected. Hematological markers, including the HALP score, systemic immune-inflammation index (SII), neutrophil-to-lymphocyte ratio (NLR), monocyte-to-lymphocyte ratio (MLR), and platelet-to-lymphocyte ratio (PLR), were calculated. The severity of HEG was assessed using the Pregnancy-Unique Quantification of Emesis and Nausea (PUQE) score. Body mass index, platelet count, lymphocyte count, and albumin levels were significantly lower in the HEG group, whereas NLR, MLR, and SII were significantly higher (*p* < 0.05 for all). The HALP score was also significantly lower in patients with HEG and demonstrated a strong negative correlation with disease severity. Both NLR and SII were positively correlated with PUQE scores. ROC analysis revealed that NLR and MLR had moderate diagnostic accuracy for HEG. In the multivariable logistic regression analysis, only NLR was identified as an independent risk factor for HEG development. The results indicate that hematological inflammatory indices, particularly the NLR and HALP score, are significantly altered in HEG. Although their discriminative ability was only moderate, they may be considered easily accessible and supportive indicators rather than stand-alone diagnostic tools. Further prospective multicenter studies are needed to confirm the clinical utility of these biomarkers in the management of hyperemesis gravidarum.

## Introduction

Hyperemesis gravidarum (HG) is a serious clinical condition characterized by severe nausea and vomiting during pregnancy^1^. This disorder can lead to systemic complications, such as dehydration, ketonuria, and weight loss, by disrupting the maternal fluid and electrolyte balance. Unlike the physiological process limited to mild nausea and morning sickness, HG is a significant pregnancy complication that reduces the quality of life and increases the need for medical care^2,3^.

Although the exact etiopathogenesis of HG remains unclear, various mechanisms related to hormonal, genetic, neurovegetative, and immune factors are thought to contribute to this condition^4^. This complex interplay not only complicates early diagnosis but also leads to significant variability in individual responses^5^. Therefore, there is an increasing need for practical, reliable, and objective biomarkers that can help predict the disease course.

In modern medicine, systemic inflammation and immune responses play critical roles in the diagnosis, prognosis, and therapeutic monitoring of numerous diseases^6,7^. In this context, hematological markers derived from complete blood count parameters have gained prominence in clinical practice because of their widespread availability and low cost^8^. Although several hematological indices, such as NLR, PLR, and SII, have previously been associated with HG, the HALP score, which integrates hemoglobin, albumin, lymphocyte, and platelet values, has been investigated in only a limited number of studies^9,10^. HALP reflects not only systemic inflammation but also nutritional status^11^, and is particularly relevant in HG, where both mechanisms are critically involved.

Therefore, this study aimed to evaluate the HALP score in relation to the presence and severity of HG, along with other hematologic inflammatory indices. In doing so, this study sought to investigate the potential clinical value of these parameters in contributing to the diagnosis and management of HG.

## Materials and methods

### Study design and setting

This prospective observational study was conducted between March and June 2025 at the Department of Obstetrics and Gynecology, Kütahya City Hospital, Turkey. Pregnant women diagnosed with hyperemesis gravidarum were compared to healthy pregnant controls matched for gestational age.

### Participants and eligibility criteria

A total of 99 pregnant women were enrolled in this study. The study group comprised 48 women between 6 and 14 weeks of gestation who were diagnosed with hyperemesis gravidarum. The control group included 51 low-risk healthy pregnant women matched for gestational age. Written informed consent was obtained from all participants before enrollment. The inclusion criteria were as follows: maternal age between 18 and 40 years, singleton intrauterine pregnancy between 6 and 14 weeks of gestation, diagnosis of hyperemesis gravidarum based on clinical and laboratory findings (for the study group), absence of major obstetric complications at the time of enrollment, and availability of complete clinical and laboratory data. The exclusion criteria were as follows: presence of gastrointestinal or metabolic disorders; chronic systemic diseases such as diabetes mellitus, hypertension, thyroid disorders, and renal or hepatic disease; autoimmune and hematological disorders; acute or chronic infections; use of immunosuppressive, anti-inflammatory, or immunomodulatory medications; multiple pregnancies; molar or ectopic pregnancies; known fetal or maternal anomalies; history of blood transfusion within the previous three months; and incomplete clinical or laboratory records.

The diagnostic criteria for hyperemesis gravidarum (HG) were defined as persistent vomiting occurring more than twice per day, weight loss of greater than 5% of the pre-pregnancy body weight, and the presence of at least one positive ketonuria result. The modified pregnancy-unique quantification of emesis and nausea (PUQE) scoring system was used to assess the severity of HG. Each participant completed a three-item questionnaire evaluating nausea, vomiting, and retching experienced during the first trimester^12^. Based on PUQE scores ranging from 4 to 15, patients with HG were classified as mild (≤ 6 points), moderate (7–12 points), or severe (≥ 13 points). Accordingly, the study population was divided into three categories: mild (*n* = 5), moderate (*n* = 26), and severe (*n* = 17).

### Data collection

Fasting venous blood samples were collected from all participants for the analysis of complete blood count (CBC) parameters. The measured laboratory variables included hemoglobin, albumin, lymphocyte, platelet, neutrophil, and monocyte counts. The Pregnancy-Unique Quantification of Emesis and Nausea (PUQE) scoring system was employed to assess the severity of hyperemesis gravidarum, which evaluates the frequency of nausea, vomiting, and retching over a 12-hour period. Based on the PUQE scores, participants were categorized into three groups: mild (4–6 points), moderate (7–12 points), and severe (≥ 13 points). All clinical and demographic data were recorded using standardized data collection forms.

### Calculation of biomarkers

Based on the complete blood count data, several hematologic inflammatory indices were calculated, including the HALP score, Systemic Immune-Inflammation Index (SII), neutrophil-to-lymphocyte ratio (NLR), monocyte-to-lymphocyte ratio (MLR), and platelet-to-lymphocyte ratio (PLR). The HALP score was calculated by multiplying hemoglobin, albumin, and lymphocyte values, and then dividing the result by the platelet count: HALP = (Hemoglobin × Albumin × Lymphocyte)/Platelet. SII was calculated as: SII = (Platelet × Neutrophil)/Lymphocyte. NLR, MLR, and PLR were calculated as follows: NLR = Neutrophil/Lymphocyte, MLR = Monocyte/Lymphocyte, and PLR = Platelet/Lymphocyte.

### Ethical approval

This study was reviewed and approved by the Non-Interventional Clinical Research Ethics Committee of Kütahya University of Health Sciences. Ethical approval was granted on May 6, 2025 (decision number 2025/06 − 01). This study was conducted in accordance with the principles of the Declaration of Helsinki. All participants provided written informed consent prior to enrollment, and the confidentiality of personal data was strictly maintained throughout the study.

### Statistical analysis

Statistical analyses were performed using IBM SPSS Statistics for Windows (version 27.0; IBM Corp., Armonk, NY, USA). The distribution of continuous variables was evaluated using the Shapiro–Wilk test. Continuous variables with a normal distribution are presented as mean ± standard deviation (SD), while categorical variables are expressed as numbers and percentages (%). The choice of statistical tests was based on the distribution and types of data. Continuous variables with normal distribution were compared using the independent samples t-test, whereas the Mann–Whitney U test was applied for variables that were not normally distributed. Categorical variables were analyzed using Pearson’s chi-square or Fisher’s exact tests, as appropriate. Correlations between hematological and inflammatory parameters and disease severity (PUQE score) were assessed using Pearson or Spearman correlation analyses, depending on the distribution of the variables. To evaluate the diagnostic performance, receiver operating characteristic (ROC) curves were constructed for NLR and MLR, and the area under the curve (AUC), sensitivity, specificity, and optimal cut-off values were calculated. ROC analysis was selected as the most appropriate method to evaluate the discriminative ability of the hematological indices. Multivariable logistic regression analysis was performed to determine the independent risk factors for hyperemesis gravidarum. Variables with *p* < 0.10 in the univariate analysis were included in the regression model to reduce the risk of overfitting and identify independent predictors. A p-value < 0.05 was considered statistically significant. Post-hoc power analysis was performed using G*Power (v3.1.9.7, Düsseldorf, Germany). For a two-tailed independent-samples t-test with α = 0.05, n₁=51 (controls) and n₂=48 (HEG), and Cohen’s d = 0.68 (for NLR; SD_pooled 3.1; d=(5.3–3.2)/3.1), the achieved power was 0.92 (df = 97).

## Results

The baseline demographic and obstetric characteristics of the study population are shown in Table 1. The mean age of the participants was 27.6 ± 4.8 years, and the mean body mass index (BMI) was 25.7 ± 5.3 kg/m². The mean gravidity and parity were 1.9 ± 1.2 and 0.7 ± 0.9, respectively. The mean number of living children was 0.7 ± 0.8, and the mean number of abortions was 0.2 ± 0.5. The average gestational age at the time of inclusion was 9.9 ± 3.8 weeks. Fourteen participants (14.1%) reported active smoking, and 13 (13.1%) had a history of hyperemesis gravidarum (Table 1).

**Table 1:** Baseline demographic and obstetric characteristics of the study population.

	Mean±S.D.
Age (years)	27.6±4.8
BMI (kg/m²)	25.7±5.3
Gravida (n)	1.9±1.2
Parity (n)	0.7±0.9
Living Children (n)	0.7±0.8
Number of Abortions (n)	0.2±0.5
Gestational Age (weeks)	9.9±3.8
Smoking Status, *(n*,*%)*	14 (14.1)
History of HEG, *(n*,*%)*	13 (13.1)

Continuous variables are presented as mean ± SD, and categorical variables as n (%). Abbreviations: BMI, body mass index; HEG, hyperemesis gravidarum; SD, standard deviation.

The mean age was lower in the HEG group (26.4 ± 4.5 years) than in the control group (28.7 ± 4.9 years), although this difference did not reach statistical significance (*p* = 0.095). The mean BMI was significantly lower in the HEG group (24.9 ± 4.9 kg/m²) than in the control group (26.4 ± 5.7 kg/m²; *p* = 0.013). Gravida, parity, number of living children, number of abortions, gestational age, and smoking status did not differ significantly between the groups (*p* > 0.05 for all). Notably, a previous history of HEG was significantly more common in the HEG group (27.1%) than in the control group (*p* < 0.001) (Table 2).

**Table 2:** Comparison of demographic and obstetric characteristics between HG and control groups.

	Control (*n* = 51)	HG (*n* = 48)	*p* value
	Mean±S.D.	Mean±S.D.	
Age (years)	28.7±4.9	26.4±4.5	0.095^a^
BMI (kg/m²)	26.4±5.7	24.9±4.9	0.013 ^a^
Gravida	2.0±1.2	1.8±1.1	0.288^b^
Parity	0.8±0.9	0.6±0.9	0.170 ^b^
Living Children	0.8±0.9	0.5±0.8	0.123 ^b^
History of Delivery, *NSD (n %)*	15 (53.6)	9 (47.4)	0.676 ^c^
Number of Abortions	0.2±0.5	0.2±0.5	0.341 ^b^
Gestational Age (weeks)	9.5±3.5	10.4±4.0	0.225 ^b^
Smoking Status, *(n %)*	8 (15.7)	6 (12.5)	0.649 ^c^
History of HEG, *(n %)*	0 (0.0)	13 (27.1)	**< 0.001** ^d^

Continuous variables are expressed as mean ± standard deviation (SD), and categorical variables are presented as numbers (n) and percentages (%). Statistical tests used: a = independent samples t-test, b = Mann–Whitney U test, c = Pearson’s chi-square test, d = Fisher’s exact test. Abbreviations: HEG, hyperemesis gravidarum; BMI, body mass index; NSD, normal spontaneous delivery; SD, standard deviation.

**Fig. 1:**
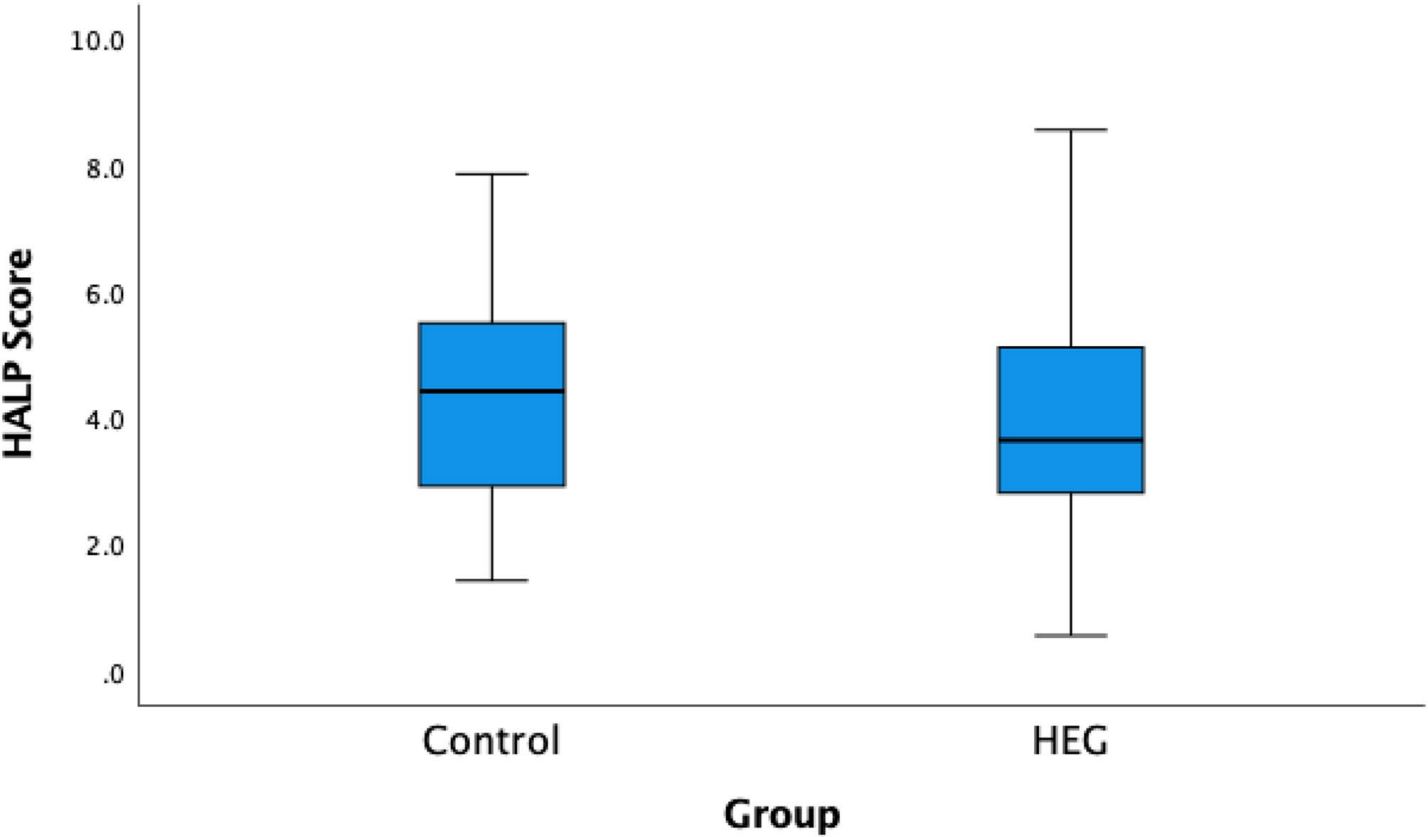
Distribution of HALP Score between the Control and HEG groups presented as boxplots.

The platelet count was significantly lower in the HEG group (259.3 ± 64.3 × 10³/µL) than in the control group (287.0 ± 70.3 × 10³/µL; *p* = 0.043). Lymphocyte count was also significantly reduced in the HEG group (1.8 ± 0.8 × 10³/µL vs. 2.2 ± 0.6 × 10³/µL; *p* = 0.023), while albumin levels were lower as well (42.6 ± 3.6 g/dL vs. 43.9 ± 2.4 g/dL; *p* = 0.035). Among the inflammatory indices, NLR (*p* = 0.006), MLR (*p* = 0.004), SII (*p* = 0.046), and HALP score (*p* = 0.011) showed statistically significant differences between the groups. The NLR and MLR were elevated in the HEG group, whereas the HALP score was decreased. There were no statistically significant differences in hemoglobin levels, monocyte count, neutrophil count, or PLR values between the groups (*p* > 0.05 for all) (Table 3; Fig. 1).

**Table 3:** Comparison of hematologic and inflammatory parameters between HG and control groups.

	Control (*n* = 51)	HG (*n* = 48)	*p* value	Effect Size
	Mean±S.D.	Mean±S.D.		
Hemoglobin (g/dL)	12.5±1.3	12.7±1.3	0.347^a^	Cohen’s d = − 0.19 (95% CI: − 0.59, 0.20)
Platelet (10³/µL)	287.0±70.3	259.3±64.3	**0.043** ^**a**^	Cohen’s d = 0.41 (95% CI: 0.01, 0.81)
Lymphocyte (10³/µL)	2.2±0.6	1.8±0.8	**0.023** ^**a**^	Cohen’s d = 0.47 (95% CI: 0.07, 0.86)
Monocyte (10³/µL)	0.6±0.2	0.6±0.2	0.971^a^	Cohen’s d = 0.01 (95% CI: − 0.39, 0.40)
Neutrophil (10³/µL)	6.4±2.2	7.2±2.8	0.145^a^	Cohen’s d = − 0.29 (95% CI: − 0.69, 0.10)
Albumin (g/dL)	43.9±2.4	42.6±3.6	**0.035** ^**a**^	Cohen’s d = 0.43 (95% CI: 0.03, 0.83)
NLR	3.2±1.3	5.3±4.2	**0.006** ^**b**^	*r* = − 0.27 (Rank-biserial)
MLR	0.31±0.17	0.43±0.31	**0.004** ^**b**^	*r* = − 0.29 (Rank-biserial)
PLR	143.6±55.0	183.4±127.7	0.196^b^	*r* = − 0.13 (Rank-biserial)
SII	909.0±397.5	1304.9±970.2	**0.046** ^**b**^	*r* = − 0.17 (Rank-biserial)
HALP Score	4.4±1.6	3.9±1.8	**0.011** ^**a**^	Cohen’s d = 0.28 (95% CI: − 0.12, 0.67)

Comparison of hematologic and inflammatory parameters between hyperemesis gravidarum (HG) and control groups. Continuous variables are expressed as mean ± standard deviation (SD). Statistical tests used: a = independent samples t-test; b = Mann–Whitney U test. Effect sizes with 95% confidence intervals are reported in parentheses. Abbreviations: HG, hyperemesis gravidarum; NLR, neutrophil-to-lymphocyte ratio; MLR, monocyte-to-lymphocyte ratio; PLR, platelet-to-lymphocyte ratio; SII, systemic immune-inflammation index; HALP, hemoglobin × albumin × lymphocyte/platelet. Effect sizes are reported with 95% confidence intervals: Cohen’s d for parametric tests and rank-biserial correlation lymphocyte/platelet tests.

Significant positive correlations were observed between PUQE score and NLR (*r* = 0.688, *p* < 0.001), MLR (*r* = 0.559, *p* < 0.001), PLR (*r* = 0.675, *p* < 0.001), and SII (*r* = 0.689, *p* < 0.001). In contrast, a significant negative correlation was found between the PUQE and HALP scores (*r* = − 0.728, *p* < 0.001) (Table 4).

**Table 4:** Correlation between PUQE score and inflammatory markers in HEG Patients.

		PUQE Score
NLR	Correlation	0.688
	p value	**< 0.001**
MLR	Pearson Correlation	0.559
	Sig. (2-tailed)	**< 0.001**
PLR	Pearson Correlation	0.675
	Sig. (2-tailed)	**< 0.001**
SII	Pearson Correlation	0.689
	Sig. (2-tailed)	**< 0.001**
HALP Score	Pearson Correlation	−0.728
	Sig. (2-tailed)	**< 0.001**

Pearson’s correlation coefficients (r) are presented for each biomarker. All p-values < 0.05 were considered statistically significant and are shown in bold. Abbreviations: NLR, neutrophil-to-lymphocyte ratio; MLR, monocyte-to-lymphocyte ratio; PLR, platelet-to-lymphocyte ratio; SII, systemic immune-inflammation index; HALP, hemoglobin × albumin × lymphocyte/platelet; PUQE, Pregnancy-Unique Quantification of Emesis and Nause.

**Fig. 2:**
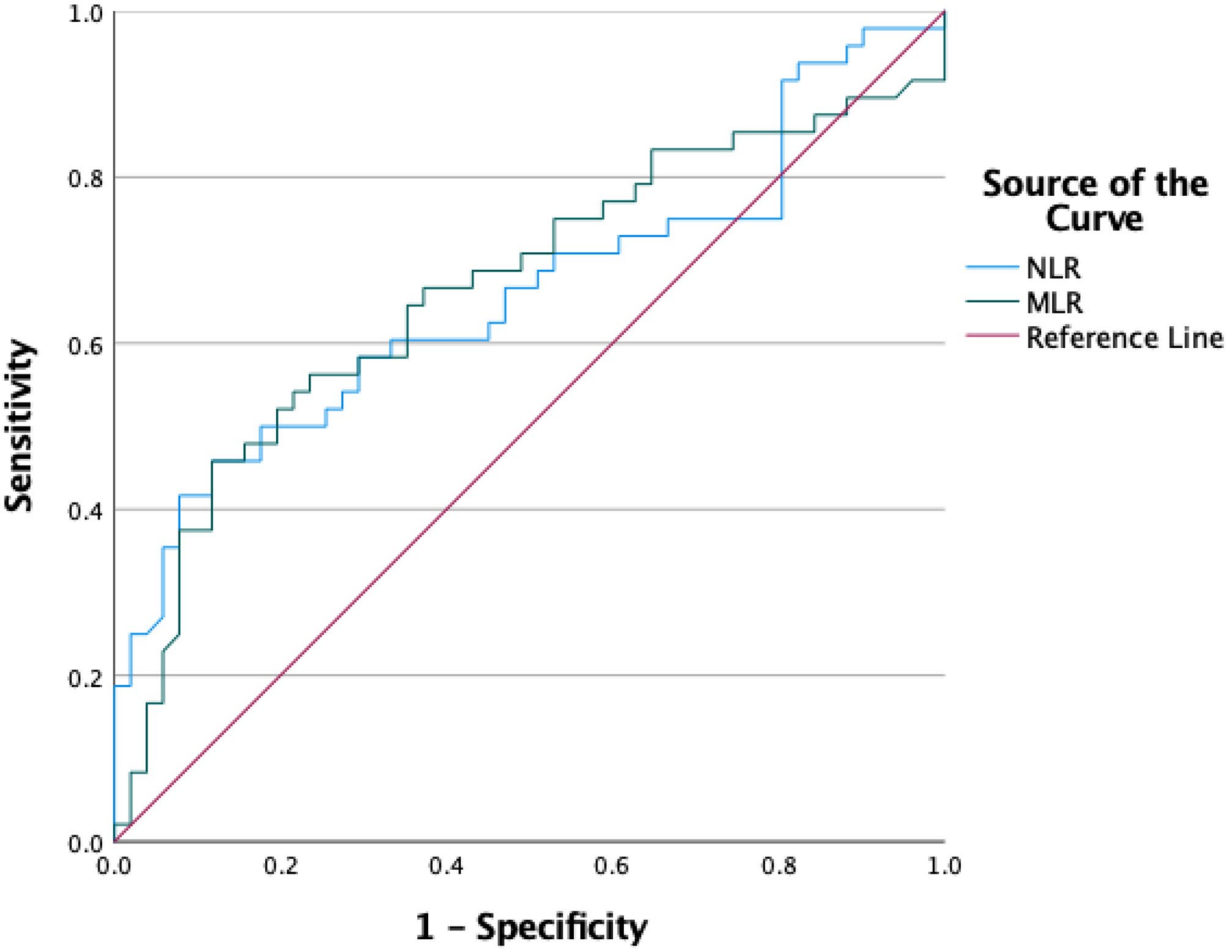
ROC curves of NLR and MLR for predicting hyperemesis gravidarum.

Both markers demonstrated moderate diagnostic performances. The area under the curve (AUC) was 0.659 for NLR (*p* = 0.006; 95% CI: 0.549–0.769) with an optimal cut-off value of 3.43, yielding 65% sensitivity and 63% specificity. For MLR, the AUC was slightly higher at 0.666 (*p* = 0.005; 95% CI: 0.555–0.776) with a cutoff of 0.3, achieving 67% sensitivity and 62% specificity (Table 5; Fig. 2).

**Table 5:** ROC curve analysis of NLR and MLR for predicting hyperemesis gravidarum.

	AUC	Sensitivity	Specificity	Cut-off	*p* value	Asymptotic 95% CI	
						Lower B.	Upper B.
NLR	0.659	65%	63%	3.43	**0.006**	0.549	0.769
MLR	0.666	67%	62%	0.3	**0.005**	0.555	0.776

Statistically significant p values are shown in bold. Abbreviations: AUC, area under the curve; CI, confidence interval; NLR, neutrophil-to-lymphocyte ratio; MLR, monocyte-to-lymphocyte ratio.

Subgroup analyses according to disease severity (based on PUQE scores) revealed a clear gradient in hematological and inflammatory indices. As shown in Table 6, the NLR, MLR, PLR, and SII values progressively increased from mild to severe HEG, whereas the HALP score significantly decreased in parallel with disease severity (all *p* < 0.001) (Table 6).

**Table 6:** Comparison of hematologic and inflammatory indices according to hyperemesis gravidarum severity (mild, moderate, severe) based on PUQE Score.

	Mild (*n* = 5)	Moderate (*n* = 26)	Severe (*n* = 17)	*p* value
	Mean±S.D.	Mean±S.D.	Mean±S.D.	
NLR	2.1±0.7	3.7±1.8	8.8±5.1	**< 0.001** ^**f**^
MLR	0.26±0.11	0.32±0.12	0.64±0.42	**< 0.001** ^**f**^
PLR	84.4±10.3	129.9±27.5	294.5±160.9	**< 0.001** ^**f**^
SII	461.2±167.2	932.1±412.4	2123.2±1154.4	**< 0.001** ^**f**^
HALP Score	7.2±0.8	4.4±0.9	2.1±0.8	**< 0.001** ^**e**^

Continuous variables are expressed as mean ± standard deviation (SD). Statistical tests used: e = one-way ANOVA; f = Kruskal–Wallis test. Abbreviations: HEG, hyperemesis gravidarum; PUQE, Pregnancy-Unique Quantification of Emesis and Nausea; NLR, neutrophil-to-lymphocyte ratio; MLR, monocyte-to-lymphocyte ratio; PLR, platelet-to-lymphocyte ratio; SII, systemic immune-inflammation index; HALP, hemoglobin × albumin × lymphocyte/platelet.

In the multivariable logistic regression analysis, only the neutrophil-to-lymphocyte ratio (NLR) remained a statistically significant and independent predictor of hyperemesis gravidarum (HG) (OR: 2.019; 95% CI: 1.048–3.889; *p* = 0.036). This finding indicates that the NLR may serve as an independent risk factor for the development of HG. Other inflammatory markers, including MLR (*p* = 0.538), PLR_100 (*p* = 0.829), SII_100 (*p* = 0.429), and HALP score (*p* = 0.421), were not significant in the multivariable model (Table 7).

**Table 7:** Multivariable logistic regression analysis results of inflammatory markers for predicting hyperemesis gravidarum.

	B	S.E.	Wald	*p* value	Odds Ratio	95% C.I.	
						Lower	Upper
NLR	0.703	0.334	4.415	**0.036**	2.019	1.048	3.889
MLR	−1.456	2.362	0.380	0.538	0.233	0.002	23.875
PLR_100	0.200	0.700	0.046	0.829	1.002	0.988	1.015
SII_100	−0.100	0.100	0.626	0.429	0.999	0.997	1.001
HALP Score	0.189	0.235	0.648	0.421	1.208	0.762	1.915
Intercept	−2.396	1.750	1.875	0.171	0.091		

OR: Odds Ratio; CI: Confidence Interval; S.E.: Standard Error. PLR and SII were scaled per 100-unit increase, MLR was scaled per 0.1-unit increase, and all other variables were modeled per 1-unit increase. Abbreviations: OR, odds ratio; CI, confidence interval; SE, standard error; NLR, neutrophil-to-lymphocyte ratio; MLR, monocyte-to-lymphocyte ratio; PLR, platelet-to-lymphocyte ratio; SII, systemic immune-inflammation index; HALP, hemoglobin × albumin × lymphocyte/platelet.

## Discussion

In this study, we obtained significant findings by evaluating the HALP score and various hematological inflammatory markers in pregnant women diagnosed with hyperemesis gravidarum (HEG). The body mass index, platelet and lymphocyte counts, and albumin levels were significantly lower in the HEG group. In contrast, inflammatory response parameters such as NLR, MLR, and SII were markedly elevated, whereas the HALP score was decreased in this group. The results also demonstrated a significant relationship between disease severity and inflammatory markers; in particular, NLR, MLR, PLR, and SII correlated positively with HEG severity, whereas the HALP score showed an inverse relationship. In the multivariable analysis, only NLR was identified as an independent risk factor for HEG development. These findings suggest that systemic inflammation may play an important role in the pathogenesis of HEG and that hematological inflammatory indices, especially NLR, may provide supportive information in the diagnosis and management of the disease. However, their discriminative performance was only moderate, suggesting that they should not be considered stand-alone diagnostic tools. Furthermore, subgroup analyses based on PUQE scores confirmed a stepwise increase in NLR, MLR, PLR, and SII with disease severity, accompanied by a significant decrease in HALP score. Although only a limited number of studies have examined the relationship between HG and inflammation in the context of the HALP score, to our knowledge, this is the first study to investigate the association between disease severity and these hematological parameters in patients with HEG.

Prompt recognition and management of hyperemesis gravidarum (HG) are essential for reducing both maternal and fetal morbidity and mortality. Inflammatory and immunological factors are believed to contribute significantly to the pathogenesis of HG. The involvement of pregnancy-related hormones, humoral factors, and various cytokines in the development of HG has been extensively discussed. Several studies have identified a significant association between elevated levels of specific cytokines (including TNF-α and IL-6), mediators (such as vaspin and sirtuin), and acute-phase reactants (like CRP) with the onset of systemic inflammation and subsequent HG^13–16^. In addition, alternative pathogenic pathways and etiologies have been proposed. The prevailing view is that uterine hypoxia during early gestation may be a contributing factor. Recent research has demonstrated that HG pathogenesis is linked to subclinical inflammation and increased oxidative stress. Nevertheless, current evidence does not clearly establish whether inflammation is a primary driver of HG or a secondary consequence^16,17^.

The HALP score, which integrates hemoglobin, albumin concentration, lymphocyte count, and platelet count, has been employed to predict prognosis in a variety of malignancies as well as in stroke. This recently recognized index serves as a significant biomarker for assessing both systemic inflammation and nutritional status^18^. Serum albumin is a primary indicator of nutritional health and visceral protein production, and its concentration is subject to change in conditions such as severe HG or hepatic disorders. In patients with HG, profound weight loss, alongside electrolyte and metabolic imbalances, may lead to maternal malnutrition. Consequently, hypoalbuminemia and anemia often emerge as complications of inadequate nutrition^19^. As the severity of HG escalates, malnutrition becomes more pronounced, resulting in further declines in the albumin levels. If symptoms remain uncontrolled, adverse fetal outcomes such as low birth weight, intrauterine growth restriction, preterm birth, congenital anomalies, and even fetal mortality may occur^20^.

Yıldırım et al. (2024) reported that inflammatory indices, such as NLR, PLR, and SII, were significantly associated with both the presence and severity of HEG, and that these parameters were identified as independent risk factors^21^. Similarly, in our study, NLR, MLR, and SII values were significantly higher in the HEG group and showed a positive correlation with disease severity. Furthermore, our study is the first to demonstrate that the HALP score was significantly lower in the HEG group and showed a negative correlation with disease severity. Our findings are consistent with the literature and suggest that the HALP score may be useful in the evaluation of HEG.

Bayram et al. (2023) demonstrated that the HALP score was significantly lower and the SII index was higher in the hyperemesis gravidarum (HEG) group. Furthermore, a decrease in the HALP score and an increase in the SII index were found to be negatively and positively associated with disease severity, respectively^9^. The authors also reported that a HALP score below 3.5 could predict HEG with a sensitivity and specificity of 78.1% and 77.6%, respectively. Similarly, in our study, the HALP score was significantly lower and the SII index was significantly higher in the HEG group. In both studies, the decrease in HALP score and the increase in SII index were associated with disease severity, indicating that these biomarkers may be useful in predicting the presence and severity of HEG.

In a study by Beser et al. (2024), the systemic immune-inflammation index (SII) was reported to be positively correlated with disease severity in patients with hyperemesis gravidarum (HEG), and it was significantly increased with higher degrees of ketonuria^22^. However, the authors emphasized that the sensitivity and specificity of SII in predicting the severity of HEG were limited; thus, its use alone in clinical practice may not be sufficient. Similarly, in our study, the SII was significantly higher in the HEG group and was positively correlated with disease severity. However, in the multivariate analysis, SII was not identified as an independent risk factor. Both studies indicate that the SII index is associated with the presence and severity of HEG; however, further studies are needed to clarify whether SII can be used as a sole diagnostic or prognostic marker in clinical practice.

In a large-sample study by Cendek et al. (2024), HALP and PNI scores were found to be significantly lower in the hyperemesis gravidarum (HEG) group, indicating that these parameters may be useful in the diagnosis of HEG^23^. In addition, the area under the curve (AUC) value for the HALP score was reported as 0.625, with a cut-off value of 35.8, and the sensitivity and specificity were 59.7% and 59.5%, respectively. The study also noted that the m-HALP score did not show a significant difference between the HEG and control groups. Similarly, in our study, the HALP score was significantly lower in the HEG group and negatively correlated with disease severity. Both studies demonstrated that the HALP score was significantly lower in HEG; however, its sensitivity and specificity were limited, indicating that HALP may serve only as a supportive biomarker rather than as a definitive diagnostic tool.

In a study conducted by Dal et al. on patients with preeclampsia, the HALP score was significantly lower in the patient group, whereas the uric acid and uric acid–creatinine ratios were higher^24^. In recent years, a growing body of research has investigated the diagnostic value of inflammatory parameters in hyperemesis gravidarum and other obstetric complications. In another study by Dal et al., the serum delta neutrophil index (DNI) and other hematological inflammatory parameters were evaluated in patients with HEG; however, only neutrophil count and NLR were reported to be significantly higher in the HEG group^25^. This finding supports our results, emphasizing that NLR is a prominent marker for the diagnosis of HEG and determining disease severity.

This study had several limitations. First, it was conducted at a single center with a relatively small sample size, which limits generalizability of the results. Additionally, owing to the study design, only women in early pregnancy (6–14 weeks of gestation) were included; therefore, findings from different gestational ages could not be assessed. Furthermore, only hematological inflammatory markers and serum albumin levels were evaluated, while other biochemical, hormonal, and oxidative stress parameters were not investigated. All data were obtained at a single time point; therefore, dynamic changes in these parameters throughout pregnancy could not be observed. Finally, as the study cohort consisted solely of Turkish women, the findings may not be directly generalizable to other ethnic or geographic populations. Therefore, larger multicenter prospective studies are needed to confirm these results. Nevertheless, the prospective design of our study, the simultaneous evaluation of multiple hematologic inflammatory indices, and the demonstration of the HALP score as being associated with HEG severity for the first time provide a novel contribution to the literature, despite these limitations.

In this study, hematological inflammatory markers, such as the HALP score, SII, NLR, MLR, and PLR, showed significant changes in pregnant women with hyperemesis gravidarum. In particular, the HALP score has emerged as a valuable biomarker for both the presence and severity of disease. The HALP score, which integrates hemoglobin, albumin, lymphocyte, and platelet counts, provides a comprehensive reflection of both systemic inflammation and maternal nutritional status. Our results indicate that a decrease in HALP is strongly associated with greater disease severity, highlighting its potential as a novel and clinically relevant biomarker for the management of HEG. Our findings support that hematological parameters and derived inflammation indices may serve as practical and easily applicable tools for the diagnosis and follow-up of hyperemesis gravidarum. Nevertheless, further validation in larger populations and multicenter prospective studies is required before these biomarkers can be clinically implemented.

## Data Availability

The datasets generated and/or analyzed during the current study are not publicly available due to local ethical restrictions regarding patient privacy and confidentiality but are available from the corresponding author upon reasonable request.
